# Application of prechop technique in phacoemulsification for cataract patients with highly liquefied vitreous: a retrospective study

**DOI:** 10.1186/s12886-022-02392-0

**Published:** 2022-04-14

**Authors:** Jing Zhao, Zhouyue Li, Yu Liu, Xiaotong Han, Shengsong Huang

**Affiliations:** 1 State Key Laboratory of Ophthalmology, Zhongshan Ophthalmic Center, Sun Yat-Sen University, 54 South Xianlie Road, Guangzhou, 510060 China; 2 Department of Medical Statistics and Epidemiology, School of Public Health, Sun Yat-Sen University, Guangzhou, 510080 China

**Keywords:** High myopia-related cataract, Post-vitrectomy cataract, Prechop technique, Phacoemulsification

## Abstract

**Background:**

Phacoemulsification using phaco-chop technique has many challenging features in cataract patients with highly liquefied vitreous. This study aimed to compare the intraoperative parameters and safety between prechop technique and traditional phaco-chop in phacoemulsification for these patients.

**Methods:**

A total of 54 eyes of 54 patients with high myopia-related or post-vitrectomy cataract that underwent phacoemulsification combined with intraocular lens implantation were included in this retrospective study. Of them, 25 eyes that received manual prechop were included in the prechop group, and 29 eyes with best match of age, axial length and nuclear opalescence (NO) that received standardized phaco-chop were included as the control group. The intraoperative complications and surgery parameters were compared between groups.

**Results:**

No surgical complications were observed in the prechop group, while 2 eyes with posterior capsular rupture and 1 eye with a broken ciliary zonule (10.3%) were found in the control group. There was no significant difference in phaco time, average energy, and cumulative dissipated energy (CDE) between groups (all *P* > 0.05), but for hard nuclear cataracts with NO grading ≥ 5, prechop group required less phaco time (*P* = 0.008) and CDE (*P* = 0.029). There were significant correlations between phaco time vs. NO (*r* = 0.762 vs. 0.581, both *P* < 0.005) and CDE vs. NO (*r* = 0.717 vs. 0.668, both *P* < 0.001) in the prechop group and control group, respectively.

**Conclusions:**

The prechop technique which seemed to have less intraoperative complications, reduced phaco time and CDE compared to standardized phaco-chop might be a good alternative for cataract patients with highly liquefied or vitrectomized vitreous, especially those with hard nuclear cataracts.

## Introduction

Nuclear sclerotic cataract is most common type of cataract that forms or accelerates in eyes with high myopia or after vitrectomy surgery [1–4]. The vitreous body is highly liquefied in these patients. Phacoemulsification using phaco-chop technique has many challenging features caused by the intraocular structural changes in these kinds of patients, such as increasing nuclear density, weakened zonules, fragile and more mobility of the posterior capsule due to the absence of vitreous support. These may increase the difficulty and risk of the surgery [1, 5–9].

Challenges of phacoemulsification in eyes with highly liquefied or vitrectomized vitreous include posterior bowing of lens-iris diaphragm induced by infusion pressure and consequent deepening of anterior chamber, which in turn cause the capsular bag and lens to move back. It increases the difficulty of operation since the operator can only operate by rotating the phaco probe more vertically and chop hook deep into the eye in this situation. Furthermore, as the phaco probe penetrates the eye, the infusion fluid flushes the iris from back to front, which then cause intraoperative pupillary miosis. These can further increase the difficulty of the procedure and the risk of posterior capsule rupture, especially during the process of chop. To solve this problem, Li et al. [5] reported a method of balancing the pressure of the anterior and posterior chamber by using a syringe with a flushing needle to inject balanced salt solution into the posterior chamber via the gap between the iris and the anterior capsule of the lens. However, this method also has substantial risk for several reasons below. In this procedure, pressure of anterior chamber and vitreous cavity can only be balanced for a short time because fluid injected into the vitreous cavity will also flow out through the gap of suspensory ligament, especially in cases of hard nucleus. Consequently, in order to achieve a stable balanced pressure, multiple injections of water are needed, which increase the number of times the surgical instruments must move in and out and cause a discontinuous procedure. Yu et al. [10] reported a modified technique, with phacoemulsification in the anterior chamber, to deal with post-vitrectomy cataracts. Nevertheless, this procedure requires a relatively big capsulorrhexis margin which may affect the stability of intraocular lens (IOL) in the capsular bag. Therefore, we attempted to determine if there were a safe and effective method to perform phacoemulsification in the anterior chamber, especially with white cataracts.

First described by Akahoshi in 1998 [11], the prechop technique has been used and improved by many other surgeons along with the design and introduction of new instruments [12–16]. This technique was demonstrated to be an excellent method for treating hard nucleus cataract because it significantly reduces intraoperative ultrasound energy and effective phacoemulsification time (EPT), thus reducing the loss of corneal endothelium and injuries to other intraocular structures [17]. In addition, it was also reported to be a preferred choice for cataract with abnormal suspensory ligaments of the lens [15]. In this retrospective study, we reported the clinical application of prechop for high myopia-related or post-vitrectomy cataract patients during phacoemulsification.

## Methods

### Participants

A total of 54 eyes from 54 patients were included in this retrospective study. The inclusion criteria were summarized as follows: 1) Adult patients with high myopia-related cataracts or post-vitrectomy cataracts, who underwent phacoemulsification combined with IOL implantation by one experienced surgeon (S.H) between April 2017 and January 2020; 2) All eyes received phaco-chop or manual prechop to split the nucleus before phacoemulsification; 3) All surgeries were performed through clear cornea temporal incision. The exclusion criteria included a history of ocular trauma, corneal diseases, or other ophthalmic surgeries. If a patient received bilateral surgery, only data from the left eye was included for analysis. Of them, 25 eyes that received manual prechop to split the nucleus before phacoemulsification were included in the prechop group, and 29 eyes that received phaco-chop [18] during cataract surgery were included in the control group. All patients were fully informed of the possible risks of the surgery after finishing preoperative medical examinations.

This study protocol was approved by the Institutional Review Boards of Zhongshan Ophthalmic Center, Sun Yat-sen University and conformed to the tenets of the Declaration of Helsinki.

### Medical examinations

Patients received routine preoperative ocular examinations. Age and gender were obtained from medical records due to the retrospective nature of this study. The ocular biometric data including axial length (AL), anterior chamber depth (ACD), and keratometry (K) were measured preoperatively using IOL Master (Carl Zeiss Meditec, Inc.). Surgical complications and the following parameters were included for analysis: preoperative nuclear opalescence (NO) scores, phaco time, average energy, and cumulative dissipated energy (CDE). The intraoperative parameters of eyes with intraoperative complications were not analyzed since they were not representative of the majority. According to the study by Smith [19], the nuclear hardness was closely related to NO scores. Preoperative NO was graded using the Lens Opacities Classification System III (from 0.1 to 6.9) [20].

### Prechop technique

All cataract surgeries were performed under topical anesthesia. A 2.2 mm temporal clear cornea incision was made after topical anesthesia. Ophthalmic viscosurgical device (OVD) Amvisc (Bausch & Lomb) was injected into the anterior chamber to increase tissure stabilization and to protect corneal endothelium as well. After continuous curvilinear capsulorrhexis and hydrodissection, a Sinskey hook was introduced to the anterior chamber through the main incision and engaged into the anterior pole of the nucleus. Phaco chopper was positioned to the equator of the nucleus through the side incision and pulled toward the center. The two worked together bimanually to split the nucleus into two hemisphere and then rotated the fragments by 90 degrees. The procedure was repeated twice to further divide the two hemispheres into four quadrants, followed by phacoemulsification to remove the lens fragments using Centurion® Vision System (Alcon).

### Statistical analysis

Data analysis was performed using SPSS 23.0. The two-sample Student's *t*-test and *χ*^2^ test were used to compare the demographics and characteristics between patients in control group and prechop group. To compare the intraoperative and postoperative parameters, Mann–Whitney *U* test was applied because non-normality of these variables was detected by Shapiro–Wilk test. Correlations between the NO scores and phaco time as well as the average energy and CDE were assessed using Spearman's rank correlation coefficients. All the statistical tests were two tailed and *P* values less than 0.05 were considered to be significant.

## Results

### Demographics

The baseline characteristics of the study patients before the surgery are shown in Table 1. Both age (*P* = 0.696) and gender (*P* = 0.753) were best match between the two groups. No significant difference between groups were detected for AL, ACD, mean value of steep and flat keratometry (K_mean_), preoperative visual acuity (VA) and NO scores (all *P* > 0.05). Twenty eyes in the control group and twenty-three eyes in the prechop group had high myopia with refractive error over -6.0 D or axial length over 26.0 mm. Eleven eyes in the control group and eight eyes in the prechop group had history of vitrectomy for various reasons including rhegmatogenous retinal detachment, vitreous hemorrhage, proliferative diabetic retinopathy, epimacular membrane, and macular hole defects (Table 2). The time duration between vitrectomy and cataract surgery was larger than 3 months for these patients. Four eyes in the control group and six eyes in the prechop group had high myopia and vitrectomy, and three eyes had previously received corneal refractive surgery. No silicone oil-filled eyes were included.

**Table 1:** Patient characteristics before the surgery

Parameter	Control group	Prechop group	*P* value
Eyes (n)	29	25	-
Sex, n (%)			0.753
Male	14 (48)	11 (44)	
Female	15 (52)	14 (56)	
Age (y)			
Mean ± SD	58.03 ± 10.03	57.40 ± 13.10	0.696
Median	59	54	
Range	38, 76	34, 89	
AL (mm)			
Mean ± SD	27.49 ± 3.22	28.08 ± 2.55	0.671
Median	28.15	28.49	
Range	21.58, 31.87	23.15, 34.12	
ACD (mm)			
Mean ± SD	3.39 ± 0.43	3.28 ± 0.76	0.677
Median	3.45	3.3	
Range	2.62, 4.09	3.02, 4.21	
K_mean_ (D)			
Mean ± SD	43.64 ± 2.04	43.09 ± 2.72	0.958
Median	43.76	44.04	
Range	37.88, 47.98	36.18, 45.99	
UDVA (logMAR)			
Mean ± SD	1.38 ± 0.46	1.45 ± 0.45	0.571
Median	1.30	1.52	
Range	0.40, 2.30	0.60, 2.00	
NO grading			
Mean ± SD	4.8 ± 1.2	4.6 ± 1.3	0.455
Median	4.6	4.7	
Range	2.2, 6.5	2.5, 6.7	

*SD* standard deviation, *AL* axial length, *ACD* anterior chamber depth, *K*_*mean*_ mean value of flat and steep keratometry, *UDVA* uncorrected distance visual acuity, *NO grading* nuclear opalescence grading with LOCS III, *logMAR* logarithm of minimum angle of resolution

**Table 2:** Primary indications for the 19 patients with a history of vitrectomy

Indication for vitrectomy	No. of eyes
Control group	11
rhegmatogenous retinal detachment	5
vitreous hemorrhage	2
epimacular membrane	3
proliferative diabetic retinopathy	1
Prechop group	8
rhegmatogenous retinal detachment	5
macular hole	2
epimacular membrane	1

### Outcome and complications

At postoperative day one, 86.2% (25/29) eyes in the control group and 92% (23/25) eyes in the prechop group showed improved uncorrected distance visual acuity (UDVA). We observed significant increase in UDVA after the surgery in both groups (Both *P* < 0.001). Intraoperative complications occurred in three eyes in the control group while no complication was observed in the prechop group. Of them, 2 eyes had a history of vitrectomy due to rhegmatogenous retinal detachment, and 1 eye had a history of vitrectomy because of epimacular membrane before cataract surgery. Of the 2 eyes which had posterior capsular rupture because of posterior capsule touch during cataract surgery, one underwent successful phacoemulsification, and the other had the nuclei dropped into the vitreous chamber. For the latter, pars plana vitrectomy was conducted to remove the nuclear fragments. One eye had a broken ciliary zonule compromising over 50% of the circumference during cataract surgery. Two of the three complicated eyes had adequate capsular support for a sulcus-based IOL implantation. Implantation failed in one eye because of inadequate capsular support. No corneal endothelial decompensation, endophthalmitis, or other serious ocular complications were observed.

### Analyses of intraoperative parameters

As mentioned above, three eyes that had intraoperative complications in the control group were not included for analyses of intraoperative parameters. Two Of the three eyes had NO grading less than 5, and the other had NO grading ≥ 5. The phaco time and energy use of the other 51 patients in both groups are shown in Table 3. IOL power, UDVA, phaco time, average energy, and CDE showed no significant difference between the control and prechop group. However, for hard nuclear cataracts with NO grading ≥ 5, prechop group had less phaco time (*P* = 0.008) and CDE (*P* = 0.029) as compared with the control group (shown in Table 4). The average energy use in both groups was not significantly different. For patients with NO grading less than 5, the intraoperative parameters did not show a significant difference between the two groups (all *P* > 0.05).

**Table 3:** Comparison of intraoperative and postoperative parameters

Parameter	Control group	Prechop group	*P* value
Phaco time (s)	48.5 ± 28.3	39.9 ± 18.5	0.376
Average energy (%)	19.7 ± 5.8	19.1 ± 5.5	0.770
CDE	10.1 ± 7.2	7.7 ± 4.2	0.366
IOL power (D)	12.5 ± 6.5	12.0 ± 5.5	0.842
UDVA (logMAR)	0.87 ± 0.54	0.63 ± 0.47	0.087

Data are expressed as mean ± standard deviation

*CDE* cumulative dissipated energy, *IOL* intraocular lens, *UDVA* uncorrected distance visual acuity, *logMAR* logarithm of minimum angle of resolution

**Table 4:** Effective phacoemulsification time in different nuclear opalescence (NO) grading group

Parameter	Control group	Prechop group	*P* value
NO < 5			
Eyes (n)	16	14	
NO grading	4.0 ± 0.7	3.6 ± 0.7	0.101
Phaco time (s)	32.5 ± 19.8	34.1 ± 11.5	0.448
Average energy (%)	18.6 ± 6.1	17.0 ± 4.2	0.790
CDE	6.5 ± 5.2	5.8 ± 2.6	0.637
NO ≥ 5			
Eyes (n)	10	11	
NO grading	6.2 ± 0.3	5.8 ± 0.6	0.223
Phaco time (s)	74.2 ± 19.6	47.3 ± 23.2	0.008*
Average energy (%)	21.4 ± 5.1	21.8 ± 6.0	0.973
CDE	16.0 ± 6.3	10.1 ± 4.7	0.029*

Mean ± standard deviation

*NO* nuclear opalescence, *CDE* cumulative dissipated energy

**Fig. 1:**
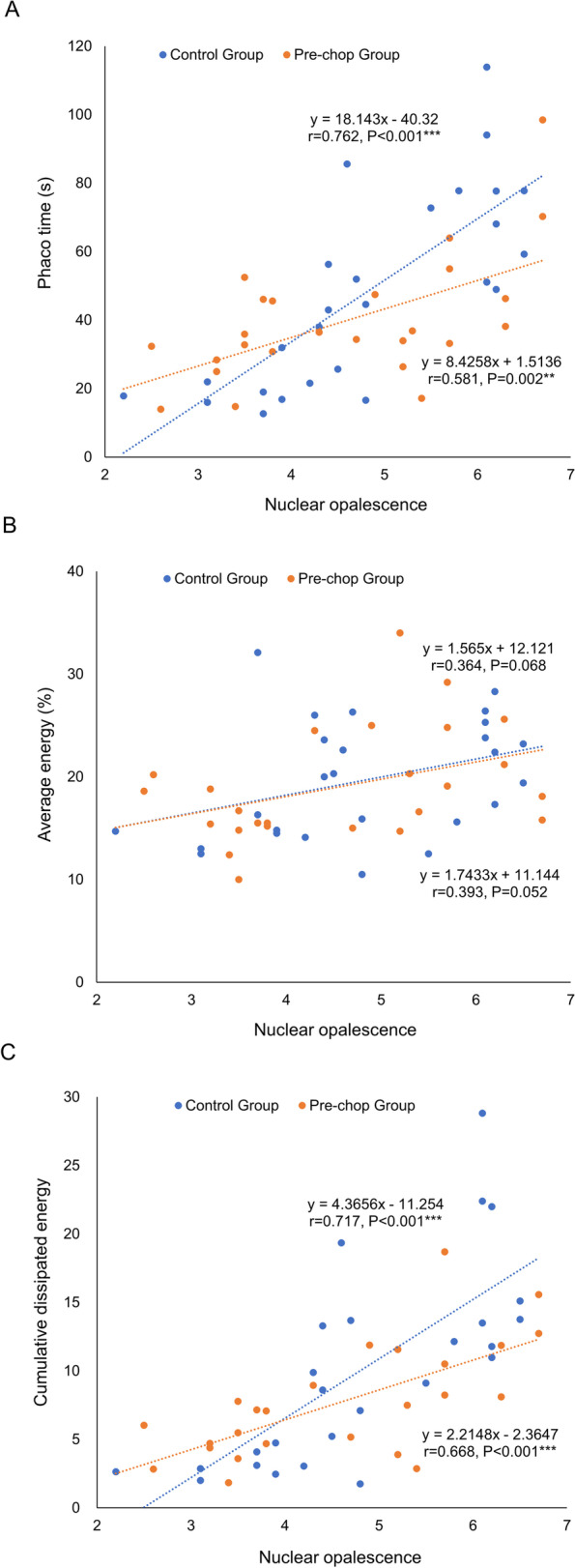
Correlation between intraoperative parameters and nuclear opalescence (NO). **A**. correlation between phaco time and NO score; **B**. correlation between average energy and NO score; **C**. correlation between cumulative dissipated energy (CDE) and NO score

Relationships between phaco time, energy, and NO are shown in Fig. 1. In the control group, phaco time (*r* = 0.762, *P* < 0.001) and CDE (*r* = 0.717, *P* < 0.001) were strongly correlated with the NO. The correlation remained but was relatively weaker in the prechop group (*r* = 0.581, *P* = 0.002 for phaco time; *r* = 0.668, *P* < 0.001 for CDE). The correlations between average energy and NO scores were similar and insignificant in both groups (*r* = 0.364, *P* = 0.068 in the control group; *r* = 0.393, *P* = 0.052 for the prechop group).

## Discussion

Cataract surgery in patients with highly liquefied or vitrectomized vitreous is challenging due to its unique anatomical structures. To solve this problem, previous researchers, such as Li et al. [5] and Yu et al. [10] have reported modified methods which had certain effectiveness in improving the safety of surgery and reducing complications, but there are still some limitations for their methods. In the current study, we reported with encouraging results that prechop technique had an advantage in treating patients with highly liquefied vitreous compared to conventional phaco chop during cataract surgery.

As demonstrated in previous studies, both vitrectomy and high myopia increase the risk of nuclear sclerotic cataracts [21, 22]. The severity of nuclear sclerosis is greater in vitrectomized eyes than typical cataract eyes [6]. Phacoemulsification for these patients showed increased risks of complications due to alterations in anatomy and showed higher dependence on the surgeon’s experience. These patients share some common anatomical features including loss of support from the vitreous body, weakened zonules, intraoperative miosis, and increased mobility of the lens-iris diaphragm during cataract surgery. In conventional phacoemulsification, notable fluctuation of ACD and movement of the posterior capsule were observed. These changes increased the difficulty of operation and risks for broken zonules and posterior capsule rupture [1, 23].

Previous studies reported the safety of conventional phacoemulsification in post-vitrectomy cataract patients [24, 25]. However, the phaco time and energy use were not thoroughly investigated. Manual prechop was recommended in recent years as an effective procedure to reduce energy use, especially for hard nucleus cataracts [17]. In this study, we found that the prechop technique is safe and effective for high myopia-related and post-vitrectomy cataract patients. For cataracts with a hard nucleus, the prechop technique is preferred because of the reduced phaco time and CDE.

Intraoperative complications occurred in three eyes in the conventional phacoemulsification group, while no complication was observed in the prechop group. A trend should be noticed that the prechop procedure showed better safety in the current study. After manual prechop to split the nucleus, the fragments of nucleus are brought to the pupil and iris plane, while the lens-iris diaphragm moves further backward. In this way, the removal of nucleus fragments by phacoemulsification is done near the pupil plane with no extra forces against the capsule and zonules. It is unnecessary for the phaco tip to bury deeply into the nucleus. Shallow penetration provides proper protection to the posterior capsule and avoids unexpected rupture. Furthermore, when the pupil iris plane diaphragm moves backward, the handpiece needs to turn vertically to perform the phaco-chop and phacoemulsification deep in the capsule. This procedure places persistent pressure on the corneal flap, which might result in decreased maneuverability and problems with the water tightness of the incision. By proper manual prechop, the phaco procedure is done at the pupil-iris plane, thus reducing the difficulty of operation and incidence of complications.

It has been reported by previous studies that prechop could reduce energy use and corneal damage in phacoemulsification for patients with age-related cataracts [26]. In this retrospective study, we analyzed the intraoperative parameters of patients with high myopia-related and post-vitrectomy cataracts. For cataracts with NO scores < 5, these two methods did not differ significantly in phaco time or energy use. But for hard nuclear cataracts with NO grading ≥ 5, less phaco time and CDE resulted from the prechop technique. The correlation between phaco time and NO or CDE scores was also weaker in the prechop group, though the difference was not statistically different. This result may be due to a small sample size. The average energy was generally controlled by the surgeon to reduce heat damage to the corneal endothelium. The correlation between average energy and NO score was weak in both groups. The results of this study indicate that, for soft nuclear cataracts both phaco-chop and manual prechop work well for phacoemulsification. For hard nuclear cataracts, the prechop technique is preferred for its reduced phaco time and CDE as well as better surgical safety.

There are some limitations in the current study. First, the sample size was relatively small, especially for hard nuclear cataracts. Further prospective studies with larger sample size are still needed. Moreover, there might be a concern over the distance to corneal endothelium when removing cataracts at the pupil-iris plane. In patients with high myopia and previous vitrectomy, the movement of lens-iris diaphragm increases with deepening of the anterior chamber. This helped keeping the phaco probe further away from the corneal endothelium. A similar technique was reported by Yu et al., who recommended phacoemulsification in the anterior chamber for post-vitrectomy cataract [10]. Another point should be noticed was that the patients included may have had poor retinas before surgery because all of them had histories of high myopia or vitrectomy surgery. Therefore, the log MAR postoperative visual acuity were poor in both groups, but these have little influence on the comparison of the intraoperative parameters and safety between prechop technique and traditional phaco-chop in phacoemulsification in the current study.

In conclusion, the results of our study are encouraging that the prechop technique exhibited a trend to have less intraoperative complications, reduced phaco time and CDE compared to standardized phaco-chop during cataract phacoemulsification surgery. It might be a good alternative for cataract surgery for patients with highly liquefied vitreous, such as in high myopia-related and post-vitrectomy cataract, especially those with hard nucleus.

## Data Availability

The datasets used and/or analyzed during the current study are available from the corresponding author on reasonable request.

## Abbreviations

NO: Nuclear opalescence
CDE: Cumulative dissipated energy
IOL: Intraocular lens
EPT: Effective phacoemulsification time
AL: Axial length
ACD: Anterior chamber depth
K: And keratometry
VA: Visual acuity
UDVA: Uncorrected distance visual acuity
